# The etiology of the association between parental nurturance and youth antisocial behavior: Evidence from a twin differences study

**DOI:** 10.1002/jcv2.12269

**Published:** 2024-08-19

**Authors:** Alaina M. Di Dio, Elizabeth A. Shewark, Daniel Thaler, S. Alexandra Burt

**Affiliations:** ^1^ Oberlin College Oberlin Ohio USA; ^2^ Michigan State University East Lansing Michigan USA

**Keywords:** antisocial behavior, genotype‐environment correlation, parenting, twin differences

## Abstract

**Background:**

Lower parental nurturance is consistently associated with higher levels of youth antisocial behavior (ASB), but the etiology of this association remains unclear. To fill this gap, we employed a twin differences approach to illuminate the environmental and genetic origins of the association between parental nurturance and children's ASB.

**Methods:**

Participants were 2060 twins (49% female) ages 6–10 from the Michigan State University Twin Registry. Parental nurturance and youth ASB were assessed using multiple measures (e.g., questionnaires, interviews) and informant reports (e.g., twins, parents, teachers). Co‐twin difference‐score correlations were analyzed separately by zygosity using specification curve analysis, an exhaustive modeling approach that examined associations across all possible specifications of the nurturance and ASB data.

**Results:**

Parental nurturance demonstrated clear, negatively signed associations with youth ASB at the individual level. However, these associations generally did not persist within twin pairs. We observed no significant twin difference correlations within monozygotic (MZ) pairs and only a handful of significant twin difference correlations among dizygotic (DZ) pairs, in which the DZ co‐twin who experienced more nurturance exhibited less ASB. Post‐hoc analyses in these data revealed that these associations differed markedly from those with harsh parenting that suggested environmental influences on youth ASB.

**Conclusions:**

These results strongly argue against a causal influence of low parental nurturance on youth ASB, and instead suggest that genetic influences and shared environmental confounds underlie their association. Further, findings strongly suggest that different parenting behaviors are associated with child ASB via different etiologic mechanisms.

**Key points**

- Lower parental nurturance is consistently associated with more youth ASB. However, the etiology of their association remains unclear.
- Our results suggest that genetic influences partially underlie the association, such that parents are more/less nurturing in response to their child's genetically influenced behavior. We also observed a role for mediation by shared familial confounds.
- These results contrast with those observed between harsh parenting and ASB in these same data, which revealed an entirely environmental effect.
- Different parenting behaviors are associated with youth ASB for different etiologic reasons. Future work should conceptualize the origins of parental harshness and nurturance as at least partially distinct.

## INTRODUCTION

The parent‐child relationship is one of the most important contributors to child development, predicting outcomes ranging from socioemotional competencies (Zhou et al., 2002) and psychopathology (Butterfield et al., 2020) to academic achievement (Gurdal et al., 2016). Although most studies have focused on harsh/negative parenting (e.g., Berthelon et al., 2020), a smaller line of research has examined positive parenting, including parental warmth and nurturance. These studies suggest that nurturance is positively associated with children's self‐esteem (Buri et al., 1992; Khaleque, 2013) and contributes to the development of socioemotional skills including empathy (Zhou et al., 2002) and emotional responsiveness (Khaleque, 2013). There is also evidence (Arim et al., 2011; Pardini et al., 2007; Pinquart, 2017) that positive parenting can guard against the development of antisocial behavior (ASB; i.e., behaviors/attitudes that transgress societal norms and infringe upon others' rights; Burt, 2012). Indeed, in a large cross‐cultural, longitudinal study, higher levels of parental warmth predicted significant decreases in ASB across ages 8–10 and 10–14 in the majority of cultures/ethnic groups examined (Rothenberg et al., 2020). In short, youth ASB appears to be less pronounced in the presence of nurturing parenting.

Researchers have frequently interpreted these results via a “parent effects model,” in which parental nurturance inhibits the development of child ASB (Collins et al., 2000; Maccoby, 2000). The use of family based, correlational designs (even longitudinal ones), however, precludes assumptions about the direction of causation. Experimental treatment research has filled this gap to some extent, typically demonstrating that improving parenting practices (i.e., developing better discipline strategies) reduces children's ASB (Bellair et al., 2019; Li et al., 2017; Odgers et al., 2012; Sampson & Laub, 1994).

Critically, however, the fact that parenting interventions can reduce ASB in treatment‐seeking families does not illuminate the origins of their association prior to treatment. Indeed, extant work has also pointed to the presence of child‐driven effects, where parents are responding to their child's ASB by becoming harsher and less warm. In one classic example, Anderson et al. (1986) examined boys (ages 6–11) with and without conduct disorder (CD) and their mothers interacting in unrelated pairs, finding that mothers of boys with CD did not differ from mothers of children without CD in negative or positive behaviors when interacting with children without CD. However, all mothers gave more commands to boys with CD than to those without, suggesting that children with CD evoked different reactions than children without CD.

Quasi‐experimental twin studies have a unique role to play in understanding the origins of associations between parenting and youth ASB. One powerful tool is the child‐based twin differences design. Monozygotic (MZ) twins are genetically identical and thus differ only as a function of their child‐specific environmental experiences (e.g., differential parenting) and measurement error. Because MZ twins share 100% of their genes, significant associations between MZ co‐twin differences in parenting and differences in their ASB necessarily point to an environmental pathway between nurturance and ASB. By contrast, differences between dizygotic (DZ) twins are attributable to child‐specific environmental factors and the 50% of their segregating genes the twins do not share (Plomin et al., 2008). Significant twin difference associations in only DZ twins would thus suggest genetic mediation of the association between parenting and youth ASB. Finally, if difference‐score associations are null in both MZ and DZ pairs, then the association is both genetic and shared environmental (i.e., influences that increase similarity between twins regardless of their genetic relatedness) in origin.

Importantly, the use of a *child‐based* twin differences design allows us to clarify the likely source of these genetic influences. Namely, genetic influences in child‐based twin designs are inferred based on the genetic relatedness of the twins who are receiving (not providing) the parenting (Klahr & Burt, 2014). As such, genetic processes reflect the influence of the child's genetic makeup on the parenting they receive, an effect referred to as an evocative gene‐environment correlation (*r*GE) (Jaffee et al., 2012). Evocative *r*GE are non‐random, genetically influenced exposures to environmental experiences, whereby children's heritable characteristics evoke reactions from others in their environment consistent with their genetic predispositions. In the case of parental nurturance and ASB, for example, parents of children with ASB may be less nurturing toward their children as they grapple with their children's misbehavior. To the extent that the children's behavior is genetically influenced, their genes have shaped their environmental experiences.

Extant twin studies have used the twin difference and other genetically informed designs to illuminate the origins of associations between parenting and youth ASB. Much of this work has pointed to environmental effects of harsh parenting on youth ASB (Burt et al., 2006, 2021; Klahr et al., 2011). For example, Burt et al. (2021) examined 1030 twin pairs (the same sample examined herein) and found that, among twins discordant for levels of harsh parenting, the twin experiencing harsher parenting exhibited more ASB, and this association was equivalent in magnitude in MZ and DZ pairs. In other words, differences in parental harshness were associated with differences in twin ASB even when those twins were genetically identical, indicating that the association between harsh parenting and youth ASB reflects an entirely environmentally mediated pathway (that could be parent‐driven, child‐driven, or both). However, other (typically smaller) studies have suggested that links between ASB and parenting may instead be genetically influenced (Ge et al., 1996; Marceau et al., 2013; O’Connor et al., 1998). As one example, Ge et al. (1996) examined 45 adopted children (ages 12–18) and found that children at higher genetic risk for externalizing psychopathology received less nurturance and harsher discipline from their adoptive parents than adoptees who were not at genetic risk.

In sum, findings from extant twin and adoption studies examining associations between parenting and youth ASB have been mixed, with some pointing to environmentally mediated and others pointing to genetically mediated associations. Critically, however, nearly all of these studies focused on parental harshness to the exclusion of positive parenting. This is problematic, as research has consistently shown that parental harshness and nurturance represent different behaviors that are only modestly associated (e.g., *r*s = −0.18 to −0.28 in O’Connor et al., 1998). Moreover, prior research has indicated that etiologic effects may differ for negative parenting dimensions versus positive ones (Klahr & Burt, 2014). As such, the links between ASB and parental nurturance may be distinct from those observed between harsh parenting and ASB, although research has yet to confirm this possibility.

There is thus a clear need for additional twin research that seeks to understand the origins of the association between ASB and nurturing parenting in particular. By elucidating the mechanisms underlying the association between nurturance and ASB, such work could inform intervention efforts aimed at reducing the prevalence of ASB. The goal of the current study was to do just this utilizing a twin differences approach. Considering the limited evidence of *r*GE for parental warmth and prior findings of environmental mediation of the links between parenting and child ASB in these (Burt et al., 2021) and other data, we hypothesized that the association between parental nurturance and child ASB would reflect an environmentally driven association.

Importantly, twin differences research, as with all research, are influenced by researcher decisions regarding the data. For example, researchers must decide which dimension of ASB to examine (e.g., aggression, or nonaggressive rule‐breaking), which informant reports to examine (e.g., mother, father, twin, etc.), whether to adjust for nonnormality in the ASB data, and so on. Different researchers can make different choices, usually for legitimate reasons. However, these decisions can alter findings and be prone to *p*‐hacking, in that significant findings may be present only under particular specifications (Simonsohn et al., 2019). Although a strength of our study is the availability of multiple measures and informant reports (see Methods), the availability of these data raises questions about which to include a priori. We addressed this issue by utilizing an exhaustive modeling approach (i.e., specification curve analysis) that conducts analyses across all reasonable data specifications and provides summaries of observed effects. In this way, we were able to disentangle genetic and environmental contributions to the association between parental nurturance and children's ASB in general and across various specifications of the data while avoiding the noise/bias introduced by different data decisions.

## METHODS

### Participants

Families were drawn from the Twin Study of Behavior and Emotional Development in Children (TBED‐C), a subsample of the population‐based Michigan State University Twin Registry (MSUTR; Burt & Klump, 2019). The TBED‐C includes a population‐based arm (*N* = 528 families; MZ *n* = 260, DZ *n* = 268), and an under‐resourced arm living in neighborhoods with higher‐than‐average poverty levels (*N* = 502 families; MZ *n* = 166, DZ *n* = 336). Consistent with recent publications, both arms were analyzed jointly for the current analyses. In both arms, participants were recruited through birth records (see Burt and Klump (2019) for descriptions of recruitment). Procedures were approved by the Institutional Review Board of Michigan State University. Children provided informed assent, whereas parents provided informed consent for themselves and their children.

TBED‐C twins were 6–10 years old (*M(SD)* age = 8.2 (1.47) in the population‐based arm and *M(SD)* age = 7.9 (1.49) in the under‐resourced arm), although 30 twin pairs turned 11 by the time they participated. Females comprised 48.7% of the sample. The racial identities of the full sample were 82% White, 10% Black, 1% Asian, 1% indigenous, and 6% multiracial. As described in Burt and Klump (2019), families in the under‐resourced (but not the population‐based) arm were more racially diverse than the local population (e.g., 14% Black and 77% White in the under‐resourced arm; local population based on the area census: 5% Black and 85% White). The twins' primary caregiver completed a physical‐similarity questionnaire to assess zygosity (Peeters et al., 1998). This questionnaire has an accuracy rate of ≥95%.

### Measures

#### Children's ASB

The Achenbach Child Behavior Checklist (CBCL; Achenbach & Rescorla, 2001) was completed separately by mothers and fathers for each twin, while the twins' teachers completed the corresponding Achenbach Teacher Report‐Form (TRF; Achenbach & Rescorla, 2001). We examined the Rule‐Breaking Behavior scale (e.g., lies, breaks rules, steals, truant; 17 items on the CBCL and 12 items on the TRF; *α* = 0.63–0.71) and the Aggressive Behavior scale (e.g., destroys people's things, fights, threatens people, argues; 18 items on the CBCL and 20 items on the TRF; *α* = 0.86–0.92). Maternal reports were available on 99% of twins (*n* = 2040), whereas paternal reports were available on 82.9% (*n* = 1707). The teachers of 119 twins were not available for assessment (e.g., because twins were home‐schooled, teacher contact information was incorrect). TRF data were available for 1549 participants and our teacher participation rate was 83%.

The corresponding Semi‐Structured Clinical Interview for Children and Adolescents (SCICA; McConaughy & Achenbach, 2001) was completed by nearly all twins (98.8%; *n* = 2035). Co‐twins were interviewed by separate interviewers. We focused on the 23‐item Aggressive/Rule‐Breaking scale (Achenbach & Rescorla, 2001; McConaughy & Achenbach, 2001) adapted from the CBCL and TRF. To assess interrater reliability, 10% of SCICA interviews were videotaped and coded by multiple interviewers (average intraclass *r* = 0.88).

Consistent with past meta‐analyses (Achenbach et al., 1987), informant reports were moderately intercorrelated (*r*s = 0.19–0.57; all *p*s < 0.01). We examined these reports separately in analyses. Because prior work indicates that each informant provides incrementally valid information due to their unique experiences with the child (De Los Reyes & Kazdin, 2005), we also averaged across informants to create three composites: (1) adult informants (mother, father, teacher), (2) family informants (mother, father, child), and (3) all informants (mother, father, teacher, child). When only one informant report was available, that report was used for the composite.

#### Parental nurturance

Parental support, closeness, and nurturance was measured via the Parent‐Child Involvement scale on the Parental Environment Questionnaire (PEQ; Elkins et al., 1997) (12 items; e.g., “I praise my child when he/she does something well”; “My child talks about his/her concerns and experiences with me”). Parents individually rated their nurturance of each twin, and twins individually rated the nurturance received from each parent. Each item was rated on a four‐point scale from *definitely true* to *definitely false* and coded so that higher scores indicated higher levels of nurturance. The PEQ was read to twins with reading levels under fifth grade to assume comprehension (assessed with a brief reading screen; Torgesen et al., 1999). The nurturance scale displayed good internal consistency reliability alphas across all individual informant reports (*α* = 0.68–0.80).

Maternal reports of nurturance were available for 2010 twins, paternal reports were available for 1697 twins, and twin reports of maternal and paternal nurturance were available for 2006 and 1944 twins, respectively. Parent reports were moderately correlated (*r* = 0.25). Twin reports of mother‐ and father‐child involvement were strongly correlated (*r* = 0.59), and so they were combined for all analyses to index the twin's overall perception of parent‐child involvement. Twin reports were modestly correlated with the corresponding parent reports (*r*s = 0.13 and 0.18 with mother and father, respectively). As above, we examined each informant individually and created composites: adult informants (mother, father) and all informants (mother, father, child).

Importantly, most twin pairs differed in the parental nurturance received, with only 3%–39% of co‐twins experiencing the same levels of nurturance according to any measure. Moreover, these co‐twin differences were relatively large in magnitude, ranging from 43% to 87% of the phenotypic standard deviation. For example, the mean co‐twin difference in the nurturance composite of all informants was 2.73 (range = 0–14.5), corresponding to approximately 77% of the overall phenotypic variability in this measure of nurturance across the sample (phenotypic *SD* = 3.56, *M* = 15.1, range = 0–22.67 after setting the scale minimum to zero). In other words, average twin differences in parental nurturance were three quarters of the magnitude of average differences among unrelated individuals in the sample.

### Analytic plan

We first confirmed the phenotypic association between nurturance and ASB at the individual level via zero‐order correlations. These analyses were conducted across the full sample, utilizing random intercepts in multilevel modeling (MLM) to account for the nested structure of the data (i.e., twins and parents were nested within families). We then computed twin difference scores (i.e., Twin A's score—Twin B's score, where twins were randomly assigned to Twin A or B), separately by zygosity, for all measures of parental nurturance and ASB, followed by the correlations between the computed difference scores. These twin difference correlations were conducted at the family level (rather than the individual level) and thus did not require the use of MLM.

**FIGURE 1:**
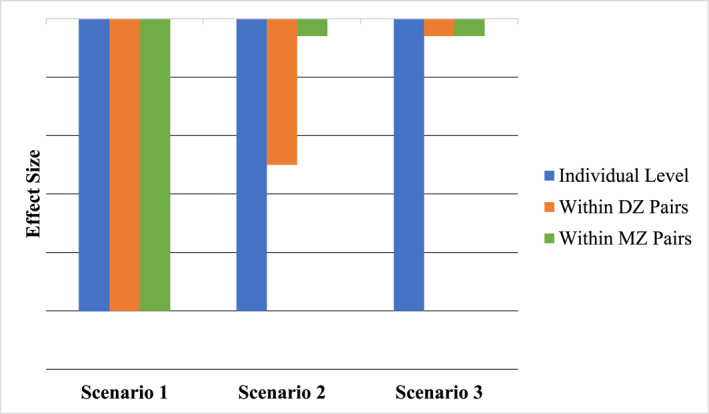
Hypothetical twin difference correlation results for the association between nurturance and ASB. In Scenario 1, the association is purely environmental in origin. In Scenario 2, the association is attributable solely to genetic influences. In Scenario 3, the association is both genetic and shared environmental in origin. DZ, Dizygotic; MZ, Monozygotic.

As discussed previously, the twin differences model is ideally suited for elucidating the origins of the association between parental nurturance and children's ASB. All twins share their overall rearing environment but differ in the amount of shared genetic material (DZ ∼ 50%; MZ = 100%). Within MZ pairs discordant for level of parental nurturance, associations control for shared environmental *and* genetic effects, thus directly measuring twin‐specific/nonshared environmental influences. If associations between parental nurturance and children's ASB are environmental in origin, we would expect to observe significant associations at the individual level and within both MZ and DZ twin pairs discordant for nurturance (see Scenario one in Figure 1; McGue et al., 2010). By contrast, if associations are observed only at the individual level and in discordant DZ twins (Scenario 2), we can infer that the association is solely genetic in origin and likely reflective of evocative *r*GE. Finally, if associations only persist at the individual level and are nonsignificant in MZ and DZ twins (Scenario 3), we would conclude that the association is due to both genetic effects (i.e., evocative *r*GE in our child‐based twin design) as well as shared environmental effects. In child twin studies of parenting, shared environmental influences can include family wide effects and confounds like maternal education, maternal mental health, family socioeconomic status, and any passive *r*GE (Klahr & Burt, 2014). Passive *r*GE reflect the fact that parents transmit their genes to their biological children while also creating their rearing environment. As such, what appears to be a causal effect of the environment can actually reflect common genes.

All analyses were conducted using a specification curve framework (Simonsohn et al., 2019), a modeling approach that avoids the noise and bias that can be introduced when selecting specific operationalizations. In this approach, researchers first identify relevant specifications (e.g., type[s] of ASB) and then generate an exhaustive combination of predictor‐outcome pairings. We focused on the following specifications: (a) ASB measurement (CBCL Aggressive Behavior scale, CBCL Rule‐Breaking Behavior scale, or the SCICA), (b) ASB informant report (mother, father, teacher, twin self report, a composite of family members, a composite of adult informants, or a composite of all informants), (c) nurturance informant reports (mother, father, twin, a composite of adult informants, or a composite of all informants), and (d) ASB normalization (skews for raw data = 1.26–3.91, while those for log‐transformed data = −0.48–1.72).

There were 130 possible specifications for the individual‐level phenotypic analyses and 260 specifications for the family‐level twin difference correlation analyses (since analyses were conducted separately by zygosity). As recommended by the specification curve developers (Simonsohn et al., 2019), we calculated median and mean effect sizes (i.e., standardized betas for MLMs and *r*s for twin difference correlations) across all specification pairings, the percentage of *p* values less than 0.05, and the median *p* value. Additionally, each *p* value was converted to a *z* score (*z* score of 1.96 corresponds to *p* = 0.05), and the average *z* score was computed. Averages were weighted by sample size to account for slightly differing sample sizes across informants. An effect was deemed statistically significantly larger than zero if neither the mean nor the median CIs included 0, the median *p* value was less than 0.05, and the average *z* score was greater than 1.96. When the four indicators did not agree, we conservatively interpreted the effect as likely nonsignificant.

All analyses were conducted in Mplus (version 8.7; Muthén & Muthén, 2021), and specification curve analyses were conducted using the Mplus automation package (Hallquist & Wiley, 2018) in *R* (version 4.1.1; R Development Core Team, 2021). Full‐information maximum‐likelihood estimation with robust standard errors was used to provide unbiased estimates for missing data (Enders, 2001). All data were standardized prior to analysis.

## RESULTS

### Phenotypic associations

Phenotypic associations across the full sample are presented in Table 1, both overall and for each of our core specifications. Parental nurturance evidenced modest, negatively signed associations with child ASB (mean and median *b*s were −0.10 and −0.11, of which 88% were significant at *p* < 0.05). These significant within‐person associations persisted across all operationalizations of children's ASB and parental nurturance, both raw and log‐transformed ASB data, and all informant reports.

**TABLE 1:** Phenotypic associations for parental nurturance.

Specification	Effect size (*b*)				
	*Mdn*	*M*	Median *p* value	% *p* < 0.05	Average *z* score
Overall	**−0.11 [−0.15, −0.06]**	**−0.10 [−0.14, −0.05]**	**<0.001**	**87.7%**	**−4.39**
Measurement of antisocial behavior					
Aggressive behavior scale	**−0.12 [−0.16, −0.07]**	**−0.11 [−0.16, −0.07]**	**<0.001**	**95%**	**−4.86**
Rule‐breaking behavior scale	**−0.11 [−0.15, −0.06]**	**−0.10 [−0.14, −0.05]**	**<0.001**	**85%**	**−4.30**
Interview	**−0.06 [−0.10, −0.02]**	**−0.05 [−0.09, −0.005]**	**0.010**	**60%**	**−2.21**
Antisocial‐behavior informant report					
Mother	**−0.11 [−0.15, −0.07]**	**−0.10 [−0.15, −0.06]**	**<0.001**	**85%**	**−4.61**
Father	**−0.10 [−0.14, −0.05]**	**−0.10 [−0.14, −0.05]**	**<0.001**	**95%**	**−4.01**
Twin	**−0.06 [−0.10, −0.02]**	**−0.05 [−0.09, −0.005]**	**0.010**	**60%**	**−2.21**
Teacher	**−0.10 [−0.15, −0.05]**	**−0.09 [−0.14, −0.04]**	**<0.001**	**80%**	**−3.51**
Family	**−0.11 [−0.15, −0.07]**	**−0.10 [−0.15, −0.06]**	**<0.001**	**90%**	**−4.65**
Adults	**−0.13 [−0.17, −0.08]**	**−0.12 [−0.17, −0.08]**	**<0.001**	**100%**	**−5.48**
All	**−0.12 [−0.16, −0.08]**	**−0.11 [−0.15, −0.06]**	**<0.001**	**90%**	**−4.89**
Nurturance informant report					
Mother	**−0.12 [−0.16, −0.07]**	**−0.11 [−0.15, −0.06]**	**<0.001**	**100%**	**−4.82**
Father	**−0.05 [−0.10, −0.004]**	**−0.06 [−0.11, −0.01]**	**0.048**	**50%**	**−2.40**
Twin	**−0.11 [−0.15, −0.07]**	**−0.11 [−0.15, −0.06]**	**<0.001**	**96.2%**	**−4.71**
Adults	**−0.10 [−0.14, −0.05]**	**−0.09 [−0.14, −0.05]**	**<0.001**	**92.3%**	**−4.00**
All	**−0.13 [−0.18, −0.09]**	**−0.13 [−0.17, −0.08]**	**<0.001**	**100%**	**−5.73**
Normalization of antisocial behavior					
Raw	**−0.10 [−0.15, −0.06]**	**−0.10 [−0.14, −0.05]**	**<0.001**	**86.2%**	**−4.20**
Log‐transformed	**−0.11 [−0.16, −0.07]**	**−0.10 [−0.15, −0.06]**	**<0.001**	**89.2%**	**−4.57**

*Note*: We report mean and median effect sizes (ES), the mean and median lower and upper 95% confidence intervals of ES in brackets, and the proportion of associations that had a *p* value < 0.05. We also converted each *p* value to a *z* score and then computed the average *z* score (*z* score = 1.96 for *p* = 0.05). To accommodate the different sample sizes across the various specifications, we weighted averages by sample size. An effect was deemed significant (indicated with bold font) only when neither set of 95% confidence intervals overlapped with zero, the median *p* value was less than 0.05, and the average *z* score was greater than 1.96. MLM results are summarized across all specifications. For example, the “Overall” row reports summary statistics across all 130 MLMs, and the row titled “Aggressive behavior scale” reports the summary statistics for the 60 MLM results containing all informant reports (both raw and log‐transformed) of the CBCL aggression measure.

### Twin difference correlations

Among MZ twins, differences in nurturance were not significantly associated with differences in their ASB (see Table 2). Within‐pair associations were small in magnitude (mean and median *r*s were −0.03 and −0.04, respectively) and only 11.5% of all specifications were significant. The median *p* value across all specifications was 0.325, the average *z* score was <1.96, and both the mean and median CIs included 0. These findings collectively indicate that MZ twin differences in parental nurturance were not associated with differences in twin ASB.

**TABLE 2:** Twin difference correlations for parental nurturance.

Specification	Monozygotic twins					Dizygotic twins				
	Effect size (*r*)		Median *p* value	% *p* < 0.05	Average *z* score	Effect size (*r*)		Median *p* value	% *p* < 0.05	Average *z* score
	*Mdn*	*M*				*Mdn*	*M*			
Overall	−0.04 [−0.14, 0.06]	−0.03 [−0.13, 0.07]	0.325	11.5%	−0.63	−0.07 [−0.15, 0.01]	−0.07 [−0.15, 0.01]	0.100	40.8%	−1.66
Measurement of antisocial behavior										
Aggressive behavior scale	−0.04 [−0.14, 0.07]	−0.02 [−0.12, 0.08]	0.311	13.3%	−0.46	−0.07 [−0.15, 0.01]	−0.08 [−0.16, 0.01]	0.104	40%	−1.85
Rule‐breaking behavior scale	−0.03 [−0.12, 0.07]	−0.03 [−0.13, 0.07]	0.411	6.7%	−0.64	−0.07 [−0.16, 0.01]	−0.07 [−0.15, 0.02]	0.087	45%	−1.63
Interview	−0.08 [−0.18, 0.02]	−0.08 [−0.18, 0.02]	0.111	30%	−1.60	−0.02 [−0.10, 0.06]	−0.03 [−0.11, 0.05]	0.378	20%	−0.74
Antisocial‐behavior informant report										
Mother	0.01 [−0.09, 0.10]	0.01 [−0.09, 0.11]	0.678	10%	0.15	−0.07 [−0.15, 0.01]	−0.08 [−0.16, 0.005]	0.087	45%	−1.87
Father	−0.03 [−0.13, 0.07]	−0.01 [−0.11, 0.10]	0.400	5%	−0.15	−0.07 [−0.15, 0.02]	−0.07 [−0.16, 0.02]	0.148	30%	−1.56
Twin	−0.08 [−0.18, 0.02]	−0.08 [−0.18, 0.02]	0.111	30%	−1.60	−0.02 [−0.10, 0.06]	−0.03 [−0.11, 0.05]	0.378	20%	−0.74
Teacher	−0.002 [−0.12, 0.11]	−0.01 [−0.12, 0.11]	0.446	0%	−0.14	−0.05 [−0.15, 0.05]	−0.06 [−0.16, 0.04]	0.315	20%	−1.17
Family	−0.07 [−0.17, 0.03]	−0.06 [−0.15, 0.04]	0.155	15%	−1.18	−0.09 [−0.16, −0.01]	−0.07 [−0.15, 0.01]	0.037	55%	−1.78
Adults	−0.02 [−0.12, 0.08]	−0.02 [−0.12, 0.08]	0.520	0%	−0.40	−0.07 [−0.15, 0.01]	−0.08 [−0.16, −0.001]	0.082	45%	−2.02
All	−0.07 [−0.17, 0.02]	−0.07 [−0.16, 0.03]	0.149	30%	−1.40	−0.09 [−0.17, −0.01]	−0.08 [−0.16, 0.01]	0.031	60%	−1.85
Nurturance informant report										
Mother	−0.05 [−0.14, 0.06]	−0.03 [−0.13, 0.07]	0.360	0%	−0.66	**−0.11 [−0.19, −0.03]**	**−0.10 [−0.18, −0.02]**	**0.008**	**73.1%**	**−2.36**
Father	0.01 [−0.09, 0.12]	0.01 [−0.10, 0.11]	0.383	15.4%	0.14	−0.02 [−0.11, 0.07]	−0.02 [−0.11, 0.08]	0.611	3.8%	−0.33
Twin	−0.05 [−0.15, 0.05]	−0.05 [−0.15, 0.05]	0.325	7.7%	−1.01	−0.05 [−0.14, 0.03]	−0.06 [−0.14, 0.03]	0.233	19.2%	−1.40
Adults	−0.02 [−0.12, 0.07]	−0.02 [−0.11, 0.08]	0.411	11.5%	−0.34	**−0.09 [−0.17, −0.01]**	**−0.09 [−0.17, −0.01]**	**0.027**	**65.4%**	**−2.14**
All	−0.06 [−0.16, 0.03]	−0.06 [−0.16, 0.04]	0.206	23.1%	−1.22	−0.07 [−0.16, 0.01]	−0.08 [−0.16, 0.005]	0.080	42.3%	−1.87
Normalization of antisocial behavior										
Raw	−0.04 [−0.14, 0.06]	−0.03 [−0.13, 0.07]	0.345	10.8%	−0.68	−0.07 [−0.15, 0.01]	−0.07 [−0.16, 0.01]	0.110	41.5%	−1.73
Log‐transformed	−0.04 [−0.14, 0.06]	−0.03 [−0.13, 0.07]	0.316	12.3%	−0.59	−0.07 [−0.15, 0.01]	−0.07 [−0.15, 0.02]	0.099	40%	−1.60

*Note*: We report mean and median effect sizes (ES), the mean and median lower and upper 95% confidence intervals of ES in brackets, and the proportion of correlations that had a *p* value < 0.05 for each specification. We also converted each *p* value to a *z* score and then computed the average *z* score (*z* score = 1.96 for *p* = 0.05). To accommodate the different sample sizes across the various specifications, we weighted averages by sample size. An effect was deemed significant (indicated with bold font) only when neither set of 95% confidence intervals overlapped with zero, the median *p* value was less than 0.05, and the average *z* score was greater than 1.96. Twin difference correlation results are summarized across all specifications separately by zygosity. For example, the “Overall” row reports summary statistics across all 130 twin difference correlations in MZ twins and summary statistics across all 130 twin difference correlations in DZ twins. The row titled “Aggressive behavior scale” reports the summary statistics separately by zygosity for the 60 twin difference correlation results in MZ twins and the 60 twin difference correlation results in DZ twins, both containing all informant reports (both raw and log‐transformed) of the CBCL aggression measure.

Similarly, among DZ twins, twin differences in nurturance were often not significantly associated with differences in their ASB. The average *z* score among DZ twins was <1.96, the median *p* value was 0.100, the correlations were modest in size (mean and median *r*s = −0.07), both the mean and the median CIs included 0, and 40.8% of all specifications were significant. There were only two exceptions to this general pattern, both involving nurturance informant reports. DZ co‐twin differences in maternal reports of nurturance and the adult informant composite of nurturance were significantly associated with co‐twin differences in twin ASB (median *p* values = 0.008 and 0.027, respectively). Additionally, and although we interpret them as nonsignificant, we note that the family, adults, and all informant composites of ASB exhibited some evidence of significance (see Table 2).

### Post‐hoc analyses: Comparison of nurturing and harsh parenting

One remaining question concerns the extent to which the findings for nurturance differ from those for parental harshness. To evaluate this question, we compared the above MZ twin difference results to those from Burt et al. (2021), which evaluated the association between ASB and harsh parenting in the same data using the same specification curve framework. This allowed for *direct, within‐person comparisons* between nurturance and harshness. In Burt et al. (2021), harsh parenting was assessed via the 12‐item Parent‐Child Conflict scale on the PEQ and a ‘hitting in anger’ item. To maximize comparisons with the current PEQ‐based assessment of nurturance, we omitted “hitting in anger” from the current analyses and focused on the PEQ Parent‐Child Conflict scale (see Supplemental Materials for information regarding this measure). Comparisons were conducted using methods developed by Lee and Preacher (2013) to test for differences between two dependent correlations that include a common variable.

Results are presented in Tables 3 and 4. Within‐MZ associations between ASB and harsh parenting were small but robustly significant (mean and median *r*s = 0.19 and 0.17, respectively), indicating that the MZ co‐twin receiving harsher parenting also engaged in more ASB. Moreover, MZ twin difference correlations between ASB and harshness were significantly larger than those for nurturance, with only a few exceptions (i.e., teacher report of ASB, father and twin reports of nurturance).

**TABLE 3:** Twin difference correlations for parental nurturance and harsh parenting in MZ twins.

Specification	Parental nurturance					Harsh parenting				
	Effect size (*r*)		Median *p* value	% *p* < 0.05	Average *z* score	Effect size (*r*)		Median *p* value	% *p* < 0.05	Average *z* score
	*Mdn*	*M*				*Mdn*	*M*			
Overall	−0.04 [−0.14, 0.06]	−0.03 [−0.13, 0.07]	0.325	11.5%	−0.63	**0.17 [0.06, 0.28]**	**0.19 [0.08, 0.30]**	**0.002**	**70.8%**	**3.45**
Measurement of antisocial behavior										
Aggressive behavior scale	−0.04 [−0.14, 0.07]	−0.02 [−0.12, 0.08]	0.311	13.3%	−0.46	**0.28 [0.15, 0.39]**	**0.24 [0.13, 0.34]**	**<0.001**	**73.3%**	**4.46**
Rule‐breaking behavior scale	−0.03 [−0.12, 0.07]	−0.03 [−0.13, 0.07]	0.411	6.7%	−0.64	**0.15 [0.04, 0.27]**	**0.14 [0.03, 0.26]**	**0.010**	**66.7%**	**2.53**
Interview	−0.08 [−0.18, 0.02]	−0.08 [−0.18, 0.02]	0.111	30%	−1.60	**0.14 [0.04, 0.24]**	**0.15 [0.04, 0.25]**	**0.007**	**80%**	**2.86**
Antisocial‐behavior informant report										
Mother	0.01 [−0.09, 0.10]	0.01 [−0.09, 0.11]	0.678	10%	0.15	**0.21 [0.08, 0.32]**	**0.20 [0.08, 0.31]**	**0.002**	**75%**	**3.53**
Father	−0.03 [−0.13, 0.07]	−0.01 [−0.11, 0.10]	0.400	5%	−0.15	**0.17 [0.06, 0.28]**	**0.17 [0.05, 0.28]**	**0.003**	**70%**	**2.88**
Twin	−0.08 [−0.18, 0.02]	−0.08 [−0.18, 0.02]	0.111	30%	−1.60	**0.14 [0.04, 0.24]**	**0.15 [0.04, 0.25]**	**0.007**	**80%**	**2.86**
Teacher	−0.002 [−0.12, 0.11]	−0.01 [−0.12, 0.11]	0.446	0%	−0.14	0.04 [−0.06, 0.16]	0.04 [−0.07, 0.16]	0.494	0%	0.77
Family	−0.07 [−0.17, 0.03]	−0.06 [−0.15, 0.04]	0.155	15%	−1.18	**0.24 [0.12, 0.34]**	**0.24 [0.13, 0.35]**	**<0.001**	**100%**	**4.47**
Adults	−0.02 [−0.12, 0.08]	−0.02 [−0.12, 0.08]	0.520	0%	−0.40	**0.20 [0.09, 0.31]**	**0.21 [0.10, 0.31]**	**0.001**	**80%**	**3.86**
All	−0.07 [−0.17, 0.02]	−0.07 [−0.16, 0.03]	0.149	30%	−1.40	**0.24 [0.13, 0.34]**	**0.24 [0.14, 0.34]**	**<0.001**	**95%**	**4.61**
Parenting informant report										
Mother	−0.05 [−0.14, 0.06]	−0.03 [−0.13, 0.07]	0.360	0%	−0.66	**0.20 [0.10, 0.30]**	**0.22 [0.12, 0.32]**	**<0.001**	**84.6%**	**4.42**
Father	0.01 [−0.09, 0.12]	0.01 [−0.10, 0.11]	0.383	15.4%	0.14	**0.15 [0.04, 0.28]**	**0.18 [0.06, 0.30]**	**0.013**	**69.2%**	**2.91**
Twin	−0.05 [−0.15, 0.05]	−0.05 [−0.15, 0.05]	0.325	7.7%	−1.01	0.05 [−0.06, 0.18]	0.08 [−0.03, 0.19]	0.419	38.5%	1.56
Adults	−0.02 [−0.12, 0.07]	−0.02 [−0.11, 0.08]	0.411	11.5%	−0.34	**0.22 [0.11, 0.33]**	**0.25 [0.14, 0.36]**	**<0.001**	**84.6%**	**4.56**
All	−0.06 [−0.16, 0.03]	−0.06 [−0.16, 0.04]	0.206	23.1%	−1.22	**0.24 [0.12, 0.35]**	**0.21 [0.09, 0.32]**	**<0.001**	**76.9%**	**3.70**
Normalization of antisocial behavior										
Raw	−0.04 [−0.14, 0.06]	−0.03 [−0.13, 0.07]	0.345	10.8%	−0.68	**0.17 [0.06, 0.29]**	**0.19 [0.07, 0.30]**	**0.002**	**70.8%**	**3.30**
Log‐transformed	−0.04 [−0.14, 0.06]	−0.03 [−0.13, 0.07]	0.316	12.3%	−0.59	**0.17 [0.06, 0.28]**	**0.19 [0.08, 0.29]**	**0.001**	**70.8%**	**3.60**

*Note*: We report mean and median effect sizes (ES), the mean and median lower and upper 95% confidence intervals of ES in brackets, and the proportion of correlations that had a *p* value < 0.05 for each specification. We also converted each *p* value to a *z* score and then computed the average *z* score (*z* score = 1.96 for *p* = 0.05). To accommodate the different sample sizes across the various specifications, we weighted averages by sample size. An effect was deemed significant (indicated with bold font) only when neither set of 95% confidence intervals overlapped with zero, the median *p* value was less than 0.05, and the average *z* score was greater than 1.96. Twin difference correlation results within MZ twin pairs are summarized across all specifications separately by parenting behavior. For example, the “Overall” row reports summary statistics across all 130 twin difference correlations for nurturance and the summary statistics across all 130 twin difference correlations for harshness. The row titled “Aggressive behavior scale” reports the summary statistics separately by parenting behavior for the 60 twin difference correlation results for nurturance and the 60 twin difference correlation results for harshness, both containing all informant reports (both raw and log‐transformed) of the CBCL aggression measure.

**TABLE 4:** Comparing dependent correlations between nurturance and harsh parenting in MZ twins.

Specification	*r*		*z* score	*p* value	*r*		*z* score	*p* value
	Nurturance	Harshness			Nurturance	Harshness		
	*Mdn*	*Mdn*			*M*	*M*		
Overall	**−0.04**	**0.17**	**−2.69**	**0.007**	**−0.03**	**0.19**	**−2.82**	**0.005**
Measurement of antisocial behavior								
Aggressive behavior scale	**−0.04**	**0.28**	**−4.16**	**<0.001**	**−0.02**	**0.24**	**−3.36**	**<0.001**
Rule‐breaking behavior scale	**−0.03**	**0.15**	**−2.30**	**0.022**	**−0.03**	**0.14**	**−2.17**	**0.030**
Interview	**−0.08**	**0.14**	**−2.81**	**0.005**	**−0.08**	**0.15**	**−2.94**	**0.003**
Antisocial‐behavior informant report								
Mother	**0.01**	**0.21**	**−2.57**	**0.010**	**0.01**	**0.20**	**−2.44**	**0.015**
Father	**−0.03**	**0.17**	**−2.56**	**0.011**	**−0.01**	**0.17**	**−2.30**	**0.021**
Twin	**−0.08**	**0.14**	**−2.81**	**0.005**	**−0.08**	**0.15**	**−2.94**	**0.003**
Teacher	−0.002	0.04	−0.53	0.594	−0.01	0.04	−0.63	0.526
Family	**−0.07**	**0.24**	**−4.00**	**<0.001**	**−0.06**	**0.24**	**−3.87**	**<0.001**
Adults	**−0.02**	**0.20**	**−2.83**	**0.005**	**−0.02**	**0.21**	**−2.96**	**0.003**
All	**−0.07**	**0.24**	**−4.00**	**<0.001**	**−0.07**	**0.24**	**−4.00**	**<0.001**
Parenting informant report								
Mother	**−0.05**	**0.20**	**−3.21**	**0.001**	**−0.03**	**0.22**	**−3.22**	**0.001**
Father	0.01	0.15	−1.79	0.074	**0.01**	**0.18**	**−2.18**	**0.029**
Twin	−0.05	0.05	−1.27	0.204	−0.05	0.08	−1.65	0.099
Adults	**−0.02**	**0.22**	**−3.09**	**0.002**	**−0.02**	**0.25**	**−3.49**	**<0.001**
All	**−0.06**	**0.24**	**−3.87**	**<0.001**	**−0.06**	**0.21**	**−3.47**	**<0.001**
Normalization of antisocial behavior								
Raw	**−0.04**	**0.17**	**−2.69**	**0.007**	**−0.03**	**0.19**	**−2.82**	**0.005**
Log‐transformed	**−0.04**	**0.17**	**−2.69**	**0.007**	**−0.03**	**0.19**	**−2.82**	**0.005**

*Note*: We report mean and median effect sizes (ES) across difference score correlation results for both parental nurturance and harshness overall and across the various specifications. For example, the “Overall” row reports the mean and median ES across all 130 twin difference correlations for nurturance and the mean and median ES across all 130 twin difference correlations for harshness. The row titled “Aggressive behavior scale” reports the mean and median ES separately by parenting behavior for the 60 twin difference correlation results for nurturance and the 60 twin difference correlation results for harshness, both containing all informant reports (both raw and log‐transformed) of the CBCL aggression measure. We also report the *p* value and the corresponding *z* score of the correlation comparison analysis. Correlations were bolded when the MZ difference correlation between ASB and nurturance differed at *p* < 0.05 from that between ASB and harshness.

## DISCUSSION

The present study investigated the etiology of the association between parental nurturance and ASB via a twin differences design using an exhaustive modeling approach. Although negative phenotypic associations were present across all informant reports and operationalizations, we saw little evidence that these associations persisted at the family level within MZ and DZ twin pairs. Put another way, co‐twin differences in the nurturance they received were rarely associated with differences in their ASB. Because MZ co‐twins differ only as a function of child‐specific environmental influences, such findings argue against an environmentally causal association of low parental nurturance on youth ASB in middle childhood. Combined with the relative absence of twin difference associations in DZ pairs, these results are consistent with Scenario 3 (see Figure 1) and strongly suggest that the association between low nurturance and child ASB reflects genetic/evocative *r*GE and familial confounds, rather than a causal effect of nurturance on youth ASB.

There were two exceptions to the overall pattern of results in DZ twins. We observed significant difference‐score associations between maternal reports of nurturance and youth ASB in DZ twins, whereby the DZ co‐twin who exhibited less ASB also received higher levels of maternal‐reported nurturance. These DZ twin associations were also observed for the adult informant composite of nurturance (i.e., maternal and paternal reports combined), but given the small number of significant results for paternal reports of nurturance, it is likely that this finding is driven by maternal reports. The nonsignificant MZ twin difference correlations for maternal nurturance, combined with the significant DZ twin difference correlations, is more consistent with Scenario 2 and indicate that child‐driven *r*GE likely underlie the association between maternal reports of nurturance and child ASB. However, they are not consistent with a role of shared environmental familial confounds. Such findings highlight the value of a specification curve framework, in which informants can be examined individually and in combination to illuminate informant‐specific etiologies.

Our finding of genetic mediation/evocative *r*GE is consistent with the small adoption study by Ge et al. (1996), which also found that genetic influences explained the relationship between parental nurturance and ASB. Moreover, our results align with prior findings of genetic and shared environmental influences on positive parenting behaviors in general. In a large‐scale meta‐analysis (*n* = 56 studies) examining the etiology of individual differences in parenting, Klahr and Burt (2014) found that 26% of the variance in the parental warmth/positivity received by twins was due to the children's own genetic influences while 39% of the variance was due to the children's shared rearing environment. Our findings build on this prior work to suggest that these evocative *r*GE and familial confounds at least partially underlie associations between positive parenting and youth ASB.

Differences between the findings of this study and those of Burt et al. (2021) also merit discussion, particularly since both studies examined the same sample, conducted the same analyses, and examined different scales on the PEQ parenting measure. Burt et al. (2021) found robust evidence that the link between harsh parenting and children's ASB was environmental in origin, as the co‐twin who received harsher parenting also engaged in more ASB, regardless of their proportion of shared genes. Our statistical comparisons indicated that MZ twin difference correlations with harshness were significantly larger than those with nurturance, results that held across nearly all specifications. Such findings suggest that parental nurturance and harshness are associated with youth ASB for different etiologic reasons. Parental harshness exerts environmentally driven effects on ASB, whereas parental nurturance appears to function via genetic factors and shared environmental confounds.

The importance of this study is augmented by the strong sampling framework (i.e., birth records), the child‐based twin differences study design, the use of an exhaustive modeling approach, and the inclusion of direct comparisons between parental nurturance and harshness in the *same* twin sample. Despite these strengths, there are several limitations. Critically, our findings are specific to middle childhood and may not extend to other developmental periods, an important limitation given that parenting behaviors change throughout children's development. For example, maternal warmth has been found to decrease as children move into adolescence (Paikoff & Brooks‐Gunn, 1991). Future research should seek to evaluate whether and how these results persist to adolescence and other developmental stages.

Additionally, our findings cannot elucidate the specific genetic and shared environmental factors that contribute to the observed associations between nurturance and ASB. There are several possibilities in this regard. Our genetic and evocative *r*GE findings could reflect pleiotropy, whereby a single gene influences multiple distinct phenotypes. In the current study, for example, the genetic factors influencing child ASB could also directly influence familial relationships. Another possibility is that the genetic factors influencing ASB may be associated with other child behaviors/phenotypes that could collectively act to elicit less nurturing parenting. In terms of shared environmental influences, in our child‐based study, familial confounds such as neighborhood characteristics, parental personality traits, or passive *r*GE (see Klahr & Burt, 2014) could be contributing to the lack of observed significant difference‐score correlations. Future analyses should seek to illuminate the specific genetic and shared environmental mechanisms underlying the associations between parental nurturance and youth ASB.

Another limitation is that the children in our study were twins. Although twins are representative of singletons on most behavioral and relational traits (e.g., Christensen & McGue, 2020), parenting two children the same age may impose additional hurdles for parents that influence the nurturance they provide. It remains unclear, however, whether or how this influenced our findings. Finally, the cross‐sectional nature of our study design and the absence of a replication sample limits the strength of our causal inferences. Although our use of a child‐based design allows us to make stronger inferences than would otherwise be possible with a cross‐sectional study, firm conclusions regarding the direction of effects between parental nurturance and youth ASB requires longitudinal or experimental data. Future work should replicate our findings in a separate longitudinal sample and extend our understanding of causality in the association between parenting and ASB via experimental studies.

## CONCLUSIONS

Despite these limitations, our findings have several important implications. First, our results argue against the notion that parental nurturance directly reduces ASB. MZ twin difference correlations were uniformly nonsignificant, suggesting that more nurturing rearing environments do not act to environmentally reduce children's ASB. Indeed, our findings strongly suggest that genetic processes and shared environmental confounds drive associations between nurturance and ASB. Importantly, our findings of genetic and shared environmental influences do not imply that interventions/treatments aimed at reducing children's ASB by increasing nurturance would be ineffective, as the epidemiological origins of an association and its clinical applications are fundamentally different questions. Instead, our findings reveal why it may have been more difficult for parents to be nurturing with their child prior to seeking treatment. Moreover, our findings underscore the importance of the role of the child in understanding parental behaviors, while also encouraging future research to disambiguate the mechanisms underlying these processes through genetically informed and other powerful (e.g., longitudinal) designs.

Second, our study provides convincing evidence that parental nurturance and harshness are associated with children's ASB for different etiologic reasons. While the association between ASB and nurturance appears to be a function of genetic processes and familial confounds, ASB's association with harsh parenting was entirely environmentally mediated. When viewed together, these findings indicate that the origins of parental nurturance and harshness should be conceptualized as at least partially distinct, and parenting theories should explicitly consider how these etiologic differences may impact youth development.

## AUTHOR CONTRIBUTIONS

**Alaina M. Di Dio:** Conceptualization; data curation; formal analysis; software; visualization; writing – original draft preparation; writing – review & editing. **Elizabeth A. Shewark:** Conceptualization; writing – review & editing. **Daniel Thaler:** Data curation; formal analysis; resources; software; validation. **S. Alexandra Burt:** Conceptualization; funding acquisition; investigation; resources; supervision; writing – review & editing.

## CONFLICT OF INTEREST STATEMENT

None of the authors report any conflicts of interest.

## ETHICAL CONSIDERATIONS

All procedures were approved by the Institutional Review Board of Michigan State University. Children provided informed assent, and parents provided informed consent for themselves and their children. The authors assert that all procedures contributing to this work comply with the ethical standards of the relevant national and institutional committees on human experimentation and with the Helsinki Declaration of 1975, as revised in 2008.

## Supporting information

Supplementary Material

## Data Availability

The data that support the findings of this study are available on request from the corresponding author. The data are not publicly available due to privacy or ethical restrictions by the study IRB.
