# Supplementary material for: The etiology of the association between parental nurturance and youth antisocial behavior: Evidence from a twin differences study
Source: JCPP Adv. 2024 Aug 19;5(2):e12269. doi: 10.1002/jcv2.12269 (PMC12159310; doi:10.1002/jcv2.12269)
Supplement: Supplementary file 1 — Supplementary Material [file JCV2-5-e12269-s001.docx]

**Supplemental Methods**

**Harsh Parenting Measure**

The Parent-Child Conflict scale on the PEQ was used to measure harsh and conflictive parenting (12 items; e.g., “My parent often criticizes me”; “I often seem to anger or annoy my parent”). Mothers and fathers individually rated their parenting of each participating twin, and twins also rated the parenting received from each of their parents. For twins with reading levels under the fifth grade (assessed via a brief reading screen; Torgesen et al., 1999), items were read to them. Each item was rated on a 4-point scale from *definitely true* to *definitely false*. The conflict scale displayed good internal consistency reliability (αs between .75 and .87 across all informant reports).

Maternal and twin reports of harsh parenting were available for 2,013 twins and paternal reports were available for 1,698 twins. Mother and father reports of harsh parenting were correlated at *r* = .30. Like for parental nurturance, twin reports of mother-child conflict and father-child conflict were moderately correlated (*r* = .64), so twin reports of parenting were combined to measure their overall perceptions of parent-child conflict. Twin reports of parent-child conflict were only modestly correlated with mother (*r* = .15) and father (*r* = .13) reports. Again, each informant was examined individually and were combined into adult (mother, father) and all informant (mother, father, child) composites.

**References**

Torgesen, J. K., Wagner, R. K., & Rashotte, C. A. (1999). Test of word reading efficiency. Austin, TX: Pro-Ed.
